# Recent Advances in Polymer-Based Immunomodulatory Nanomaterials for Wound Healing

**DOI:** 10.3390/polym18111391

**Published:** 2026-06-03

**Authors:** Ju-Ro Lee

**Affiliations:** Department of Chemical Engineering, Dankook University, Yongin 16890, Republic of Korea; leejr0515@gmail.com

**Keywords:** wound healing, immunomodulation, polymer, polymeric biomaterials, hydrogels, nanomaterials, chronic wounds

## Abstract

Dynamic interactions among cells, including immune cells, stromal cells, endothelial cells, epithelial cells, and extracellular matrix (ECM) components, are involved in the wound healing process. In chronic wounds, particularly diabetic wounds, these interactions are hampered by prolonged inflammation and excessive reactive oxygen species generation by dysregulated immune cells, bacterial infection, and impaired angiogenesis. These pathological features have shifted the therapeutic strategies from wound coverage and antimicrobial protection toward regulation of the immune microenvironment. Polymeric and hybrid materials have emerged as promising platforms for this purpose because their versatile composition, structure, degradation behavior, mechanical properties, and drug loading capacities can be widely engineered to match the dynamic requirements of wound healing, particularly in immunomodulation strategies. In this review, we focus on the major immunological barriers and potential targets in the wound healing process using polymer-based materials. Overall, this review covers recent advances, design strategies, and challenges in immunomodulatory materials including polymer-based nanoparticles, nanofibers, hydrogels, and hybrid materials for wound repair.

## 1. Introduction

Skin wound healing is an essential biological process that restores tissue integrity after injury. In normal acute wounds, repair proceeds through partially overlapping phases of hemostasis, inflammation, proliferation, and remodeling, which are orchestrated by cells in the wound microenvironment, including neutrophils, macrophages, fibroblasts, endothelial cells, keratinocytes, and extracellular matrix (ECM) components [1,2,3,4,5]. Among the repair processes, the inflammatory response is a key regulator in wound repair. Early inflammation is required for host defense, removal of damaged tissue, and initiation of repair. However, prolonged inflammatory response must be resolved and regulated in a timely manner to promote repair pathways, including angiogenesis and re-epithelialization, and to regulate ECM remodeling through myofibroblast activation for successful wound healing [2,3].

Diabetic wounds are the most clinically challenging chronic wounds because of hyperglycemia, vascular insufficiency, neuropathy, oxidative stress, impaired angiogenesis, infection susceptibility, and immune dysfunction, which hinder the establishment of a repair-favorable microenvironment [6,7,8,9,10]. These phenomena are attributed to a prolonged inflammatory state, caused by excessive reactive oxygen species (ROS), neutrophil activation neutrophil extracellular trap (NET) formation, sustained production of inflammatory cytokines and high protease activity by macrophage polarization imbalance, and defective ECM remodeling [11,12,13,14,15,16,17,18,19]. As a result, keratinocyte migration, fibroblast function, endothelial cell activity, and granulation tissue formation are impaired, which leads to delayed or incomplete wound closure.

Conventional wound dressings primarily function as physical barriers that protect the wound from dehydration, microbial contamination, and mechanical stress. Antimicrobial dressings and growth-factor-based approaches provide additional therapeutic effects, but they often do not fully address the complex immune dysfunction of chronic wounds. Increasing evidence suggests that chronic wounds should be treated not only as open tissue defects, but also as dysregulated inflammatory microenvironments [2,11,12,13,14,15,16,20,21]. Therefore, next-generation wound therapies should be designed to actively regulate immune-cell behavior, oxidative stress, bacterial burden, angiogenic signaling, and extracellular matrix remodeling in a coordinated manner. We summarized the major limitations of current and traditional wound treatments and the corresponding design requirements for next-generation wound care systems in Table 1.

Biocompatible materials are useful platforms for this purpose because they can interact with the wound microenvironment at multiple levels. Among various material platforms, polymeric and polymer-based hybrid systems have been highlighted for wound healing applications, including nanoparticles, micelles, nanofibers, hydrogels, films, foams, and injectable platforms using nature-inspired polymers such as chitosan, alginate, hyaluronic acid (HA), collagen, gelatin, dextran, cellulose derivatives, and silk fibroin [22,23,24,25,26]. In addition, synthetic polymers such as poly(lactic-co-glycolic acid) (PLGA), polycaprolactone (PCL), polyethylene glycol (PEG), polylactic acid (PLA), polyurethane (PU), and other functional polymer networks have been utilized due to their scalable fabrication. Furthermore, polymers can be integrated with other materials such as inorganic nanoparticles, lipid nanoparticles, EV, nanozymes, antimicrobial agents, or bioactive ions to generate multifunctional hybrid systems [22,23,25,26,27,28].

Importantly, the therapeutic performance of polymer-based wound materials is closely related to design parameters determined by preparation technologies. Nanoparticle formulation controls particle size, surface charge, encapsulation efficiency, and release kinetics [29,30,31]. In hydrogel systems, polymer concentration and crosslinking density determine stiffness, swelling ratio, degradation rate, and cargo-release profiles [32,33,34]. Electrospinning regulates fiber diameter, porosity, and drug loading efficiency [35,36,37,38]. These preparation-dependent parameters influence wound retention, cellular uptake, immune-cell infiltration, cytokine retention, and local delivery of immunomodulatory agents, providing a rational basis for polymer design in wound-healing applications [39,40].

Despite this progress, several challenges remain. Many studies have demonstrated accelerated wound closure, including wound area reduction, histology, and collagen deposition, but often without sufficient analysis of the underlying immune mechanisms. More detailed mechanistic insights in neutrophil activity, NET formation, macrophage polarization balance, inflammatory cytokine signaling, oxidative stress, angiogenesis, bacterial burden, and ECM remodeling in wound microenvironment are needed. In the wound-healing process, NET formation and macrophage polarization are two major immunological checkpoints that act at distinct but overlapping stages. NET formation is mainly associated with the early inflammatory phase, from a few minutes to a day, where neutrophils contribute to host antibacterial defense. However, unresolved NET accumulation can prolong inflammation and delay progression to repair [18,19]. M1/M2 macrophage polarization becomes particularly important during the transition from inflammation to the proliferative phase, a few hours to weeks, because macrophages regulate the balance between inflammation and repair [12,41]. In addition, material properties such as degradation behavior, sterilization compatibility, long-term stability, batch reproducibility, and manufacturing scalability must be considered early in the design process. These issues are particularly important for polymeric and hybrid systems, whose biological performance can be strongly influenced by polymer source, molecular weight, crosslinking density, residual reagents, degradation products, and formulation heterogeneity. In this review, we first describe the roles of major immune cell types in wound healing, particularly neutrophils and monocytes/macrophages, and then introduce polymer-based therapeutic strategies designed to regulate their pathological or reparative functions. We further discuss how polymeric systems can modulate closely associated inflammatory processes (Table 2).

**Table 1:** Shortcomings of current wound care approaches and desired characteristics of next-generation immunomodulatory wound therapies.

Current/Traditional Treatment Category	Main Function	Major Shortcomings	Desired Next-Generation Characteristics	References
Passive wound dressings	Coverage, moisture retention, protecion	Limited control of inflammation, ROS, immune-cell dysfunction, and infection	Active regulation of the wound immune microenvironment	[9,10,22,26,40]
Antibacterial dressings	Reduction in microbial burden	NET accumulation, or macrophage imbalance	Antibacterial activity combined with host-compatible immunomodulation	[20,21,22,23,24]
Growth-factor-based approaches	Stimulation of angiogenesis or tissue repair	Limited retention, short-lived activity, and insufficient control over inflammatory pathology	Sustained/local delivery coordinated with immune and repair-stage modulation	[22,28,32]
Anti-inflammatory or antioxidant treatments	Suppression of inflammatory mediators or ROS	Non-specific suppression/promotion of physiological signaling	Microenvironment-responsive and stage-adaptive modulation	[25,27]
Conventional drug delivery systems	Local or systemic therapeutic delivery	Burst release, limited targeting, insufficient retention, or systemic exposure	Local retention, controlled release, pathological cue responsiveness, and cell-specific modulation	[29,30,31]

**Table 2:** Polymer-based immunomodulatory design strategies for wound healing.

Primary Design Strategy	Design Principle	Main Immunomodulatory Function	References
Physical microenvironment modulation	Polymeric hydrogels, fibers, films, foams, and microneedles provide wound coverage, ECM-mimetic structure	Supports a repair-favorable wound environment and limits external inflammatory or microbial stimulation	[22,23,24,25,26,35,36,37,38,39,40]
Intrinsic polymer bioactivity	Polymer chemistry, charge, sulfation, conductivity, adhesiveness, directly interact with cells, inflammatory mediators	Enables chemokine sequestration, cfDNA/NET binding, antibacterial activity, or immune-cell modulation	[33,40]
Microenvironment-responsive release or activation	ROS-, pH-, glucose-, HOCl-, or enzyme-responsive bonds trigger cargo release, degradation	Provides on-demand immunomodulation in diabetic, oxidative, infected, or NET-rich wound microenvironments	[24,25,32]
Controlled cargo delivery	Nanoparticles, hydrogels, nanofibers, and films enable localized or sustained release of molecules	Regulates neutrophil activity, macrophage phenotype, angiogenesis, inflammatory signaling, and NET formation	[29,30,31,32,33,34]

## 2. Immunological Barriers to Effective Wound Repair

Early signals from wound sites recruit innate immune cells and activate resident keratinocytes, fibroblasts, endothelial cells, and stromal cells. In addition, hemostasis provides both a physical scaffold and an immunochemical framework for subsequent repair. We summarize the major immunological barriers discussed in this section in Table 3.

### 2.1. Neutrophil Persistence and NET Formation

The inflammatory phase is initially dominated by neutrophils, which are rapidly recruited within a few minutes in response to damage-associated molecular patterns, complement fragments, bacterial products, chemokines, and lipid mediators. Neutrophils phagocytose pathogens and debris, release antimicrobial peptides, generate ROS, and secrete proteases [17,18]. These functions are beneficial during early repair, but they must be tightly limited. As healing proceeds, neutrophils undergo apoptosis and are cleared by macrophages through efferocytosis [42]. Efficient removal of neutrophils is an important checkpoint for inflammatory resolution and prevents excessive tissue damage [43]. NET formation, which generates extracellular chromatin structures decorated with antimicrobial proteins and proteases, is another important mechanism linked to impaired chronic wound healing. NET accumulation results in macrophage imbalance and further inhibition of efferocytosis [44]. Diabetes has been shown to prime neutrophils toward NET formation, thereby NET-reducing strategies have been reported to improve outcomes in diabetic wound models [45].

### 2.2. Macrophage Dysfunction and Impaired Transition to Repair

Macrophage dysfunction is another distinct feature of chronic wounds. In chronic wounds, transition from M1 to M2 is delayed or incomplete due to functional dysregulation, resulting in persistent production of tumor necrosis factor alpha (TNFα), interleukin (IL) 1β, IL6, nitric oxide, and other inflammatory mediators, together with insufficient efferocytosis, angiogenic support, and ECM remodeling [11,13,16]. This distinction is important because effective therapy should restore macrophage plasticity and resolution capacity, rather than simply increasing expression of a limited set of M2 markers [46,47]. Macrophage reprogramming is one of the most extensively studied targets in immunomodulatory wound biomaterials. Materials can regulate macrophage behavior through surface chemistry, particle size, stiffness, topography, degradation products, ion release, metabolic effects, and delivery of bioactive cargos, including cytokines, nucleic acids, antioxidants, small molecules, and EV-derived factors [48,49]. The desired outcome is not merely an increase in M2 marker expression, but restoration of macrophage functions required for resolution, including efferocytosis, angiogenic support, extracellular matrix remodeling, and suppression of chronic inflammatory signaling.

### 2.3. Oxidative Stress and Cytokine-Driven Inflammatory Amplification

Excessive ROS further amplifies the inflammatory state of chronic wounds. At physiological levels, ROS are involved in microbial killing and redox signaling. However, sustained ROS accumulation damages proteins, lipids, DNA, the extracellular matrix, endothelial cells, fibroblasts, and keratinocytes. ROS also activate inflammatory pathways such as nuclear factor kappa B (NF-κB) and inflammasome signaling, increase protease activity, and promote neutrophil and macrophage dysfunction. Therefore, oxidative stress is not only a consequence of inflammation but also a mechanistic driver of failed healing. This explains why antioxidant and ROS-responsive materials are increasingly investigated as wound immunomodulatory platforms. The first major target is oxidative stress. ROS-scavenging nanoparticles, antioxidant polymers, nanozymes, and ROS-responsive hydrogels can reduce oxidative damage and suppress redox-amplified inflammatory signaling. However, ROS should not be eliminated completely, because physiological ROS contribute to antimicrobial defense and repair signaling. Therefore, polymeric systems targeting ROS in wound sites should be designed to achieve controlled redox modulation rather than indiscriminate ROS elimination. This can be achieved using polymer or catalytic nanozyme that can buffer excessive ROS and regulate local redox state [50,51,52]. This supports the use of microenvironment-responsive systems that become activated preferentially under pathological oxidative conditions, such as ROS-rich diabetic or infected wounds [30,31,32,33]. Secreted inflammatory cytokine in the wound microenvironment is another important target. Chronic wounds often show persistent activation of NF-κB, caused by IL1β, TNFα, IL6, and related inflammatory mediators. Local delivery or disposal of polymer-based nanomaterials containing inflammatory cytokine or ROS-scavenging agents, anti-inflammatory drugs, bioactive ions, RNAs, antibodies, or pathway-specific inhibitory chemicals may reduce systemic exposure while enhancing therapeutic concentration in the wound bed, altering immune cell phenotypes.

**Table 3:** Major immunological barriers to effective wound repair and corresponding material-design implications.

Immunological Barrier	Main Pathological Feature in Chronic Wounds	Effect on Wound Repair	References
Neutrophils	Prolonged neutrophil infiltration, oxidative burst, protease release, and unresolved NET formation	Maintains the wound in a non-resolving inflammatory state and induces macrophage dysfunction	[17,18,19,42,43,44,45]
Monocytes/ macrophages	Delayed or incomplete transition from inflammatory macrophage activity toward repair-supportive functions	Releases inflammatory cytokine and weakens repair processes	[11,12,13,14,15,16,46,47,48,49]
ROS/redox microenvironment	Sustained oxidative stress caused by dysregulated immune cells	Damages wound-resident cells and promotes inflammatory signaling	[25,50,51,52]
Cytokine and chemokine imbalance	Persistent inflammatory cytokine signaling and excessive recruitment of inflammatory immune cells	Amplifies immune-cell infiltration and prevents timely inflammatory resolution	[11,13,16,20,21]
Infection-associated immune stimulation	Bacterial burden and biofilm formation continuously activate innate immune responses	Couples antimicrobial stress with prolonged inflammation	[20,21,23,24]

### 2.4. Rationale for Antibacterial Polymer-Based Immunomodulatory Materials

The infection–inflammation loop is also a major therapeutic target. In chronic wounds, persistent microbial burden and sustained inflammatory activation reinforce each other. Bacterial products continuously stimulate neutrophils and excessive inflammation by neutrophils further impair antibacterial barrier and bacterial clearance. Antibacterial polymers, cationic materials, metal/polymer composites, photothermal systems, photodynamic platforms, and anti-biofilm dressings may reduce microbial burden and thereby suppress persistent innate immune stimulation. For chronic wounds, antibacterial activity should ideally be integrated with host-compatible immunomodulation, because excessive cytotoxicity or uncontrolled ROS generation can injure keratinocytes, fibroblasts, endothelial cells, and immune cells.

Overall, effective wound repair requires integrated regulation of the immune niche of the wound microenvironment. Polymer-based biomaterials are suitable for this purpose because their degradation, mechanics, porosity, adhesiveness, and release profiles can be engineered as described in the following sections.

## 3. Polymer-Based Strategies Targeting Neutrophil- and NET-Associated Inflammation in Wounds Healing

This section provides an overview of advanced strategies for modulating neutrophil-mediated immune response in wound healing. Neutrophil and NET-targeted strategies in wound healing are beneficial, because of their persistent infiltration into the wound site and their abundance during chronic inflammation [53,54,55,56,57]. Many studies have implemented neutrophil- and NET- targeted strategies: directly modulating neutrophil phenotype, including controlling neutrophil infiltration or neutrophil phenotype; inhibiting NET formation or degrading and scavenging preformed NETs [58,59]. First, burst recruitment in acute wounds or persistent infiltration in chronic wounds could be an effective therapeutic target to reduce their activation, oxidative burst, protease release, resulting in reduced inflammatory damage. Secondly, recent strategies aimed at redirecting neutrophils toward an N2-like reparative phenotype have also been explored, with the goal of promoting angiogenesis and tissue repair rather than simply suppressing neutrophil activity [60,61]. The N1/N2 neutrophil phenotype can be regarded as a functional and context-dependent state, similar to but less established than the macrophage M1/M2 framework. In general, N1-like neutrophils are associated with pro-inflammatory activities, including ROS production, protease release, NET formation, and inflammatory cytokine secretion, whereas N2-like neutrophils exhibit anti-inflammatory and pro-repair features, such as increased expression of Arg1, Ym1, CD206, VEGF, and IL-10 [62,63]. Therefore, polarizing neutrophils toward an N2-like state may support wound healing processes. However, unlike the well-established M1/M2 framework for macrophage polarization, the concept of N1/N2 neutrophil polarization remains relatively emerging in the context of wound-healing materials [64,65]. Third, NET-targeting strategies have been developed to interrupt the chronic inflammatory loop. These approaches include preventing NET formation by suppressing NETosis, degrading preformed NET structures, or scavenging extracellular NET components from the wound microenvironment.

### 3.1. Polymer Platforms for Reprogramming Neutrophil Phenotypes

Interestingly, unlike most strategies that aim to suppress neutrophil infiltration into wound sites because of their pro-inflammatory roles, some researchers found that harnessing the phenotypic plasticity of neutrophils, especially anti-inflammatory N2 neutrophils, could be an effective way to treat chronic wounds. Ghosh et al. developed a natural polymer-based nanocomposite that can be used as a laser-activated polymeric wound interface [66]. In this system, silk fibroin-gold nanorod-based polymeric nanomaterials were utilized: silk fibroin served as a biocompatible wound-contacting matrix and embedded gold nanorods conferred near-infrared-responsive photothermal properties. Exogenous histamine release from nanomaterials showed a pro-resolving neutrophilic immune response. Researchers found that Ly-6G^+^Ym-1^+^ anti-inflammatory neutrophils accumulated in wound sites over 5 times compared to Tegaderm treatment. Consequently, increasing angiogenesis, myofibroblast activation, and epithelial-to-mesenchymal transition of keratinocytes achieved 99.3% wound closure by day 7. Similarly, Gao et al. developed a neutrophil-modulatory hydrogel by incorporating the conditioned medium of N2-polarized neutrophils into a porous gelatin methacryloyl (GelMA) matrix [67]. In this study, the porous GelMA network served as a biocompatible polymeric reservoir that possesses and gradually releases neutrophil-derived wound-repair-favorable paracrine factors, enabling prolonged exposure of anti-inflammatory components to persistently infiltrating neutrophils. Released from GelMA, N2 neutrophil-derived paracrine factors not only induced capillary formation but also promoted N2 polarization of neutrophils, which was attributed to cytokines changes, including TNFα, IL1β, and vascular endothelial growth factor (VEGF). In addition, implantation of GelMA significantly reduced the number of inflammatory neutrophils, facilitating early wound closure in a mouse model. These findings suggest that modulating neutrophil phenotypes in wound sites is an effective strategy for controlling the immune microenvironment. N2 neutrophil modulation reduced inflammatory neutrophil burden, increased pro-angiogenic signaling, and improved endothelial migration and capillary formation. These outcomes indicate that neutrophils are not only inflammatory effector cells but can also serve as regulators that connect early immune responses to vascularization in wound healing.

### 3.2. ROS-Scavenging Hydrogels for Neutrophil Modulation

Tu et al. designed a multifunctional hydrogel by crosslinking poly(PEGMA-*co*-GMA-*co*-AAM) (PPGA) with hyperbranched poly-L-Lysine-modified MnO_2_ nanozymes [68]. The polymer–nanozyme hydrogel platform provided reduced oxidative stress and inflammatory cytokine release in a mouse diabetic wound model. Multifunctional hydrogel showed cationic antibacterial effects, ROS scavenging (> 90%) and oxygen generating nanozyme function, which resulted in reduced infiltration of neutrophils starting from day 3 post-injury. The hyperbranched poly-L-lysine–MnO_2_-crosslinked PPGA network enables both ROS-scavenging function and network formation, resulting in nanozyme dispersion and local retention. In addition, the hybrid design is important because PPGA provides the hydrogel network for local retention and wound coverage, whereas HBPL–MnO_2_ contributes catalytic ROS-scavenging and oxygen-generating activity, which contribute to redox-mediated modulation of neutrophil activation in wound sites over 14 days. At day 14 post-injury, over 80% of myeloperoxidase (MPO) expression was reduced, indicating that NET formation and neutrophil activation were reduced. The decrease in inflammatory neutrophils amplified anti-inflammatory effects, which was associated with M2 macrophage polarization, collagen deposition, and neovascularization.

### 3.3. Polymeric Delivery Systems for Inhibiting NET Formation

**Figure 1:**
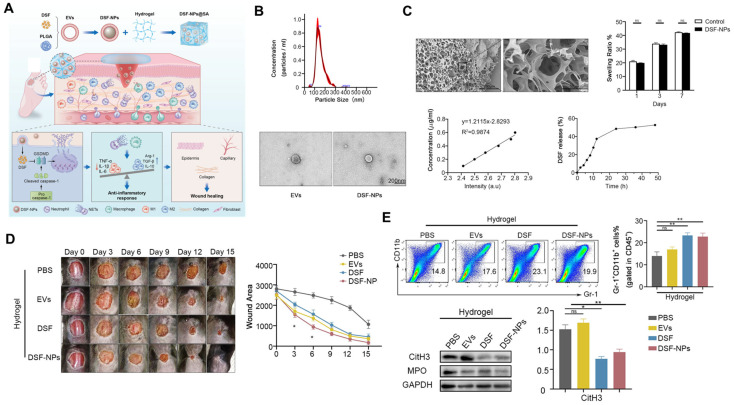
Disulfiram−loaded nanovesicle hydrogel for diabetic wound healing. (**A**) Schematic illustration of the overall therapeutic strategy using extracellular vesicle-coated, disulfiram-loaded nanoparticles embedded in a sodium alginate hydrogel for diabetic wound treatment, aimed at suppressing NET-associated inflammation and promoting wound repair. (**B**) Characterization of DSF-NPs, including particle size distribution and transmission electron microscopy images. (**C**) Characterization of the DSF-NPs@SA hydrogel, including porous microstructure, swelling behavior, calibration for DSF quantification, and drug-release behavior under high-glucose conditions. (**D**) Representative wound images and quantitative analysis of wound closure in diabetic mice treated with different hydrogel formulations. (**E**) Flow cytometric analysis of wound-infiltrating neutrophils and macrophages after treatment. * *p* < 0.05 and ** *p* < 0.01 using one-way ANOVA followed by Tukey’s test. All values are mean ± SD. ns indicates no significant difference. DSF, disulfiram; NPs, nanoparticles; EVs, extracellular vesicles; SA, sodium alginate. Scale bars are shown in the original panels. All panels reproduced from [70] under the Creative Commons Attribution 4.0 International License. Copyright © 2024 The Author(s), published by Springer Nature.

Kaur et al. utilized an alginate-GelMA hydrogel as a scaffold that incorporated the tripeptide Thr-Asp-F-amidine to inhibit protein arginine deiminase 4 to suppress excessive NETosis in diabetic wounds [69]. The alginate-GelMA polymer matrix served as a delivery platform, thereby targeting histone citrullination, a key process required for chromatin decondensation and NET formation. They confirmed that sustained release of PDA4 inhibitor from hydrogel over 72 h successfully reduced citrullinated H3 (citH3) and NET formation in human primary neutrophils in vitro. In addition, co-culture of fibroblast with PDA4 inhibitor-treated neutrophils increased fibroblast migration as well. As a result, hydrogel implantation assisted wound closure in a rat model. Sun et al. introduced EV-PLGA-disulfiram hybrid nanocomposite incorporated with sodium alginate hydrogel as a wound dressing platform (Figure 1A) [70]. This hierarchical hybrid design enables functional separation among the components: PLGA nanoparticles encapsulate the hydrophobic drug, EVs improve biological compatibility and cell surface interaction, and the alginate hydrogel provides wound retention and dressing function. The combination therefore supports sustained local NET modulation and repair-promoting immune regulation more effectively than PLGA nanoparticles alone. PLGA nanoparticles loaded with hydrophobic drug disulfiram were encapsulated in adipose stem cell-derived EVs (151.57 ± 5.92 nm), which served as both carriers of PLGA nanoparticles and immunomodulatory mediator (Figure 1B,C). Encapsulation by EVs facilitated sustained disulfiram release over 50 h. Calcium-crosslinked sodium alginate matrix, which has biocompatible, breathable, and exudate absorbable properties, maintained swelling behavior after nanocomposite loading. This hierarchical polymer-biohybrid dressing suppressed the Caspase-1/GSDMD pathway in neutrophils in wound sites. The number of Gr-1^+^CD11b^+^ neutrophils was increased 2-fold by PLGA-EV hydrogel; however, the expression of MPO was reduced by 50%, indicating reduced NET formation under high-glucose conditions in a mouse diabetic wound model (Figure 1D,E). As a result, this neutrophil modulation strategy promoted M2 macrophage polarization, angiogenesis, and re-epithelialization.

### 3.4. Hydrogel Systems for Degrading or Scavenging Preformed NETs

**Figure 2:**
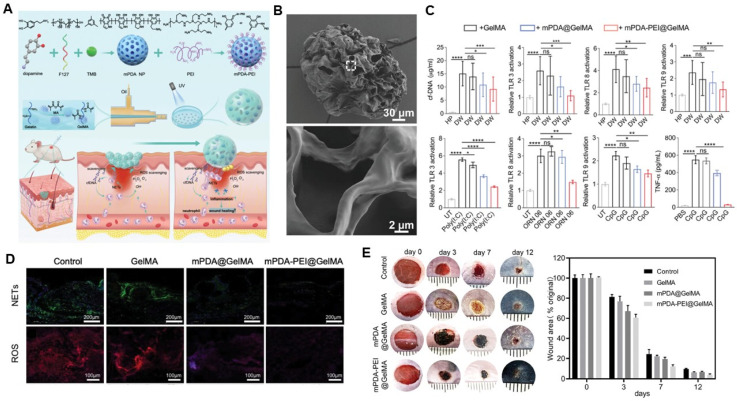
cfDNA- and ROS-scavenging mPDA-PEI@GelMA hydrogel microspheres for diabetic wound repair. (**A**) Schematic illustration of the preparation of mPDA-PEI nanoparticles and their incorporation into GelMA hydrogel microspheres, together with the proposed therapeutic mechanism involving cfDNA/NET scavenging, ROS elimination, inflammation attenuation, and improved wound healing. (**B**) Representative SEM images showing the morphology and porous microstructure of the hydrogel microspheres. (**C**) In vitro evaluation of cfDNA-binding capacity and downstream inflammatory signaling, including relative TLR activation and TNF-α production in response to different formulations. (**D**) Immunofluorescence staining of wound tissues showing NET accumulation and ROS levels after treatment with different hydrogel formulations. (**E**) Representative wound images and quantitative analysis of wound closure in diabetic wounds treated with GelMA, mPDA@GelMA, or mPDA-PEI@GelMA. GelMA, gelatin methacryloyl; mPDA, mesoporous polydopamine; PEI, polyethyleneimine; cfDNA, cell-free DNA; NETs, neutrophil extracellular traps; ROS, reactive oxygen species. * *p* < 0.05, ** *p* < 0.01, *** *p* < 0.001, and **** *p* < 0.0001 using one-way ANOVA followed by Tukey’s test. All values are mean ± SD. ns indicates no significant difference. All panels reproduced from [72] under the terms of the Creative Commons Attribution License, Copyright © 2024 The Author(s), published by Wiley-VCH GmbH.

Importantly, strategies that eliminate preformed NETs are increasingly used to treat skin wounds because neutrophils infiltrate into wound sites within a few minutes after skin injury. For example, Zhang et al. designed a hierarchical hydrogel dressing that can facilitate spatially separated drug release, including DNase I and platelet-derived growth factor (PDGF) [71]. They fabricated a nanogel with DNase I using acrylamide and azide-modified acrylamide. By combining with PDGF-containing Regranex^®^ hydrogel, PDGF is released first, while DNase I is released by exposure to hypochlorous acid later within 24 h. Without affecting the chemotaxis index of neutrophils, this hydrogel platform degraded the NET area in vitro (40%) and the number of citH3-positive neutrophils in vivo. In another study, Xiao et al. developed a hydrogel microsphere-based strategy to scavenge preformed NETs in the wound microenvironment, rather than inhibiting NET formation at the upstream signaling level (Figure 2A) [72]. They integrated GelMA and cationic polyethyleneimine (PEI)-functionalized mesoporous polydopamine. PEI functionalization in GelMA hydrogel enables the electrostatic binding of negatively charged cfDNA and NET components in wound sites. By immobilizing these extracellular inflammatory components within the hydrogel matrix, this system may limit DNA/NET-associated activation of innate immune signaling and reduce the propagation of inflammatory cytokine responses in the wound microenvironment. Xiao et al. encapsulated cationic nanoparticles in the GelMA microsphere to avoid direct nanoparticle exposure to the wound surface, like micro-cages (Figure 2B). NETs were scavenged and immobilized inside the micro-cage due to a strong binding affinity of cationic nanoparticles to cell-free DNA, reducing 50% of wound NET level (Figure 2C). This strategy does not degrade the NET backbone, thereby reducing the risk of NET-entrapped bacteria exposure. NET scavenging facilitated over 70% reduction in wound ROS level and inhibition of IL6 and TNFα (> 80%) in mouse diabetic wound sites (Figure 2D). Inflammation reduction in wound sites dramatically improved vascularization and collagen deposition, which were attributed to near-complete wound closure at day 12 (Figure 2E). Similarly, Zhong et al. designed polyacrylamide-based cationic hydrogel dressing that can scavenge not only NETs but also cell-free nucleic acids [73]. They fabricated a hydrogel with 77% of acrylamide, 20% of *N*-(3-((diemthylamino)propyl)methacrylamide, and 3% of *N*-*N*-methylenebisacrylamide, which efficiently adsorbed CpG and consequently reduced TNFα expression in RAW 264.7 cells in vitro. Five days after diabetic wound injury, the wound-implanted hydrogel adsorbed approximately six-fold more cell-free DNA, including NETs, than the control hydrogel, resulting in accelerated wound closure.

### 3.5. Polymeric Fiber and Supramolecular Nanofiber Platforms for Suppressing NET-Associated Inflammation

In the polymeric fiber platforms discussed below, NET reduction is mainly achieved through biochemical suppression of neutrophil activation and NETosis rather than physical trapping of NET structures. Minden-Birkenmaier et al. fabricated electrospun polydioxanone fiber from micrometer (1.7–2.2 µm) to nanometer (0.25–0.5 µm) scale with a bioactive additive that reduces MPO expression in neutrophils. Nanofiber with 0.1% and 1% of additive reduced NET formation and matrix metalloproteinase-9 expression 6 h after treatment [74]. In another study, Wei et al. fabricated peptide-based supramolecular nanofiber consisting of a self-assembling peptide, Nap-FFG-IGF-1 [75]. This self-assembling peptide formed a hydrogel after a heating and cooling process and showed an intertwining of nanofiber structure. Implantation of the nanofiber immediately after skin injury significantly reduced the NF-κB signaling pathway. Furthermore, at day 4 and 7 post-injury, they confirmed that the expression levels of citH3 and MPO were reduced in neutrophils within diabetic wound sites, which was attributed to NF-κB p65 pathway downregulation.

### 3.6. Enhancement of NET-Mediated Antibacterial Defense

**Figure 3:**
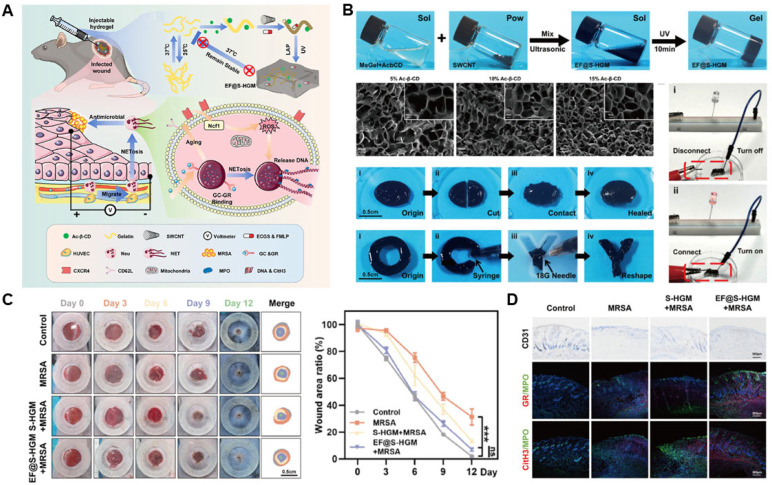
Electroactive self-healing hydrogel for antibacterial and pro-regenerative wound repair. (**A**) Schematic illustration of the injectable hydrogel strategy for infected wound treatment, showing antibacterial activity, electrical stimulation-assisted cell migration, and modulation of oxidative stress and NETosis-related inflammation. (**B**) Fabrication and characterization of the EFG-HGM hydrogel, including sol–gel transition, porous morphology, self-healing behavior, injectability, reshaping ability, and electrical conductivity. (**C**) Representative wound images and quantitative wound closure analysis in MRSA-infected wounds treated with different hydrogel formulations, with or without electrical stimulation. (**D**) Histological and immunofluorescence analyses showing angiogenesis, bacterial clearance, and NETosis-associated inflammatory responses in wound tissues after treatment. *** *p* < 0.001 using one-way ANOVA followed by Tukey’s test. All values are mean ± SD. ns indicates no significant difference. All panels reproduced from Ref. [76] under the terms of the Creative Commons Attribution 4.0 International License.

In contrast to strategies that suppress or remove excessive NETs, Ouyang et al. developed a conductive hydrogel designed to enhance NET-mediated antibacterial defense in infected wounds (Figure 3A) [76]. Researchers introduced a nanoconductive hydrogel, consisting of crosslinked acrylate-β-cyclodextrin and gelatin, through host–guest interaction (Figure 3B). They then incorporated single-walled CNTs and N-Formyl-Met-Leu-Phe, an endothelial cell growth supplement, which can induce NET formation, into the nanoconductive hydrogel. By turning on the hydrogel with an external electric current, the drugs released from the hydrogel induced NET formation of HL-60-derived neutrophils and showed antibacterial ability against S. aureus in vitro. They confirmed that nanoconductive hydrogel promoted a NET-associated physical barrier against bacterial infection, thereby limiting persistent infection-driven inflammation, resulting in improved wound closure in a mouse infected wound model over 12 days (Figure 3C,D).

Together, these studies indicate that neutrophil- and NET-modulating polymeric materials should be understood as stage-dependent immune-regulatory platforms rather than simple anti-inflammatory dressings. In acute wounds, transient neutrophil recruitment and NET-associated antimicrobial activity can support host defense, whereas in diabetic or chronic wounds, persistent neutrophil activation, oxidative burst, protease release, and NET accumulation drive tissue damage and delayed repair. Polymeric systems can intervene at multiple points by redirecting neutrophils toward reparative phenotypes, limiting excessive infiltration, inhibiting NETosis, or removing preformed NETs. In these strategies, the polymer component is essential for local retention, controlled release, wound conformability, cargo protection, and spatially restricted immune modulation. Future neutrophil-targeted materials should therefore preserve beneficial early defense while preventing the transition to chronic, NET-driven inflammation.

## 4. Polymer-Based Strategies Targeting Monocyte- and Macrophage-Mediated Inflammation in Wounds Healing

This section provides an overview of strategies for modulating monocyte and macrophage responses in wound healing process. Targeting monocytes and macrophages has been one of the most extensively studied immunological strategies for wound healing. In the wound healing process, circulating monocytes first infiltrate into wound sites within the first few days and persistently infiltrate over a week [77]. Wound-infiltrating monocytes differentiate into macrophages and orchestrate pathological reaction from start to end. During the early inflammatory phase, pathogen clearance, debris removal, neutrophil clearance, and pro-inflammatory cytokine secretion are conducted by macrophages [78]. In addition, macrophages are involved in tissue reconstruction, angiogenesis, fibroblast activation, re-epithelialization, and ECM remodeling as healing progresses. In chronic wounds, however, the macrophage phenotype should be shifted from M1 to M2 macrophages at an appropriate time point, altering the immune microenvironment in wounds, because M2 macrophages exert efferocytosis, angiogenic factor secretion, fibroblast activation, collagen deposition, and re-epithelialization, as a checkpoint for the proliferation phase. Accordingly, monocyte- and macrophage-targeted strategies using polymer-based biomaterials have been developed by many researchers: controlling excessive recruitment of monocytes and macrophage differentiation; macrophage polarization from M1 to M2 phenotype to promote tissue repair [79]; improving efferocytosis and debris clearance in the wound sites; and providing physical and biochemical cues that restore macrophage-mediated wound healing. Herein, we introduce the strategies that can modulate macrophage phenotype, one of the most abundant and powerful targets for wound healing in a manner of immunomodulation. A growing number of studies have demonstrated macrophage modulation methods with diverse cues, including cytokine and growth factor release (e.g., IL4, IL10, and PDGF), small molecule, gene, and EV delivery, and physical and material cues.

### 4.1. Chemokine-Sequestering Hydrogels for Suppressing Excessive Monocyte Recruitment

**Figure 4:**
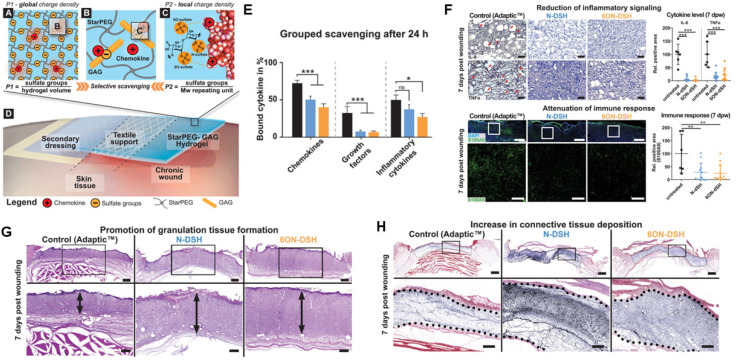
Selective chemokine-scavenging StarPEG–GAG hydrogels for chronic wound healing. (**A**–**D**) Schematic illustration of selective chemokine scavenging by StarPEG–GAG hydrogels, highlighting the effects of global and local sulfate-group density and the proposed wound dressing configuration for chronic wounds. (**E**) Grouped scavenging analysis after 24 h, showing differential binding of chemokines, growth factors, and inflammatory cytokines by the hydrogel formulations. (**F**) Histological and immunofluorescence analyses at 7 days post-wounding, showing reduced inflammatory signaling and attenuated immune responses after treatment with desulfated heparin-based hydrogel variants. Scale bars represent 1000 µm in total wound images and 250 µm in the magnified images. (**G**) Histological evaluation showing enhanced granulation tissue formation in wounds treated with the hydrogel formulations. Arrows indicate the thickness of granulation tissue. (**H**) Histological staining showing increased connective tissue deposition after hydrogel treatment. Dotted outlines indicate the connective tissue (collagen fibers). StarPEG, star-shaped poly(ethylene glycol); GAG, glycosaminoglycan; N-DSH, N-desulfated heparin; 6ON-DSH, 6-O,N-desulfated heparin. Scale bars represent 2000 µm in total wound images and 1000 µm in the magnified images (**G**,**H**). * *p* < 0.05, ** *p* < 0.01, and *** *p* < 0.001 using one-way ANOVA followed by Tukey’s test. All values are mean ± SD. ns indicates no significant difference. All panels reproduced from [83] under the terms of the Creative Commons Attribution License. Copyright © 2021 The Authors, published by Wiley-VCH GmbH.

Importantly, monocyte infiltration into wound sites and consequent inflammation after macrophage differentiation are necessary processes in wound healing [80,81]. However, prolonged and excessive inflammatory monocyte recruitment could delay wound closure and induce chronic wounds, thereby making balance control important. Therefore, chemokine scavenging or capturing materials have been widely studied as key strategies for wound healing. For example, Lohmann et al. designed a synthetic heparin-based hydrogel with glycosaminoglycan (GAG) to capture and sequester chemokines [82]. Anionic sulfate groups in GAG-functionalized hydrogel have three types of sulfates that can bind to positively charged chemokines, preventing the interaction of monocytes and chemokines that induce the inflammatory activation of monocytes. Monocyte chemoattractant protein-1 (MCP-1) and IL8 were bound to the hydrogel for up to 24 h in vitro. Implantation of GAG-hydrogel reduced Ly6C^+^Ly6G^−^ monocyte infiltration into diabetic wound sites (~40%) compared to PEG-hydrogel for 5 days. MCP-1 and IL1β cytokine levels in whole wound tissue were decreased 5 and 10 days post-injury, resulting in fibroblast activation and blood vessel formation. Similarly, Schirmer et al. developed multiarmed PEG-GAG-based hydrogel (Figure 4A) [83]. PEG-GAG hydrogel successfully captured inflammatory chemokines (Figure 4B). They also confirmed GAG-based hydrogel could capture IL8 and TNFα at day 7 post-injury, which resulted in a decrease in the number of S100A9+ inflammatory macrophages in diabetic wound sites (Figure 4C). As a result, the amount of granulation and connective tissue in wound sites was significantly increased (Figure 4D,E).

### 4.2. Microneedle-Based Spatial Control of Monocyte Trafficking

More recently, in another study, Le et al. introduced a polymeric microneedle-based chemokine capturing strategy [84]. They crosslinked a heparin/4-arm PEG-NH_2_ network onto a microneedle surface so that chemokines were spatially attracted. When the microneedle was attached onto wound sites, circulating monocytes near the wounds infiltrated into the porous microneedle rather than the wound matrix, so that nearly 75% of MCP-1 and 25% of Ly6C^+^ monocytes were captured within microneedle. After three cycles of microneedle attachment–removal, spatial monocyte population control facilitated collagen deposition, blood vessel formation, and wound closure rate improvement.

### 4.3. Selective Recruitment of Reparative Monocyte Subsets

Importantly, circulating monocytes are not phenotypically homogeneous and comprise distinct subsets, including classical, intermediate, and non-classical monocytes. They differ in chemokine receptor expression and inflammatory activity after tissue infiltration and macrophage differentiation [85,86]. In contrast to strategies that suppress excessive monocyte recruitment regardless of their phenotype, Olingy et al. designed FTY720, a small molecule that can recruit non-classical monocytes, loaded in PLGA film [87]. FTY720 is an agonist of sphingosine-1-phosphate receptor 3 that can recruit Ly6C^low^ monocytes that prefer to differentiate into M2 macrophages. After drug-loaded PLGA film implantation, the number of CD206^+^ reparative macrophages significantly increased in wound sites at day 3 post-injury.

### 4.4. Polymeric Delivery of Cytokines and Growth Factors for Macrophage Reprogramming

Kuan et al. introduced polyelectrolyte complex nanoparticles (PCNs) consisting of gelatin and alginate encapsulating VEGF, PDGF, and IL10 [88]. Encapsulation of growth factors and cytokines enabled a twofold longer release duration in vitro. They loaded IL10-encapsulated PCNs into alginate/Ca^2+^ hydrogel and implanted hydrogels in a mouse diabetic wound model. As a result, Arginase 1 (Arg1)- and CD206-positive M2 macrophages were increased and inducible nitric oxide synthase (iNOS)-positive M1 macrophages were decreased at day 14 post-injury. Moreover, mRNA expression levels of genes involved in keratinization, angiogenesis, and immune regulation were increased both at day 7 and 28. Xie et al. utilized a pH/glucose-responsive delivery strategy with phenylboronic acid (PBA)-functionalized PEI, polyvinyl alcohol, and Benzaldehyde-PEG-Benzaldehyde for the co-delivery of IL4 and salvianolic acid A [89]. Schiff base and phenylboronate ester linkages facilitate the wound microenvironment-triggered co-release of IL4 and salvianolic acid. They confirmed that ROS scavenging by salvianolic acid A and M2 macrophage polarization by IL4 changed macrophage population from M1 to M2 and cytokine profiles such as IL10, transforming growth factor beta, TNFα, and IL1β, in both in vitro and in vivo experiments.

In another study, Zhang et al. utilized prostaglandin E2 (PGE2) as a bioactive mediator of M2 macrophage transition [90]. They incorporated PGE2 into chitosan hydrogel, which resulted in the early accumulation of CD206^+^ M2 macrophages from day 1. They claimed that timepoint shift in the reparative and angiogenesis phase is the key to accelerating wound healing process.

Hydrogels provide local retention, wound-bed conformability, and sustained release of growth factors and cytokines, whereas polymeric nanoparticles improve cargo protection, co-delivery, and cellular uptake. Therefore, combining these platforms can be particularly useful when macrophage phenotype regulation requires both localized delivery and controlled presentation of cytokines or growth factors.

### 4.5. Small-Molecule-Loaded Polymer Systems for Macrophage Phenotype Modulation

Besides cytokines and growth factors, small molecules have been studied as phenotype modulators of macrophages. Li et al. used calcitonin gene-related peptide with GelMA, which is a potent vasodilator released by sensory neurons [91]. They demonstrated that wound closure rate was significantly reduced in calcitonin gene-related peptide (CGRP)-knockout mice. CGRP treatment inhibited the p53, p21, and MDM2 signaling pathway, which could promote M2 macrophage polarization. CGRP-peptide-loaded GelMA hydrogel implantation in mouse diabetic wounds augmented the expression of *Pecam1, Vegfa*, *Acta2*, and *Col2a1*, thereby accelerating wound closure.

**Figure 5:**
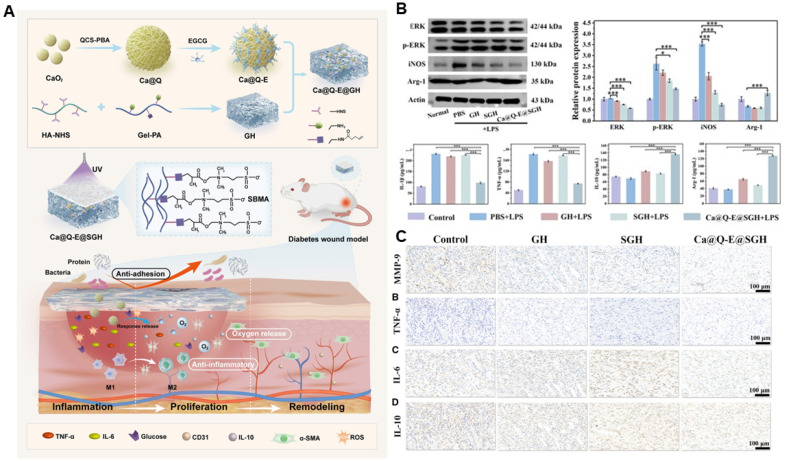
EGCG-loaded oxygen-releasing hydrogel for anti-inflammatory diabetic wound repair. (**A**) Schematic illustration of the fabrication of the Ca@Q-E@SGH hydrogel and its proposed therapeutic mechanism in diabetic wounds, including anti-adhesion, oxygen release, inflammation attenuation, macrophage polarization, and tissue remodeling. (**B**) Western blot and gene expression analyses showing regulation of ERK signaling, inflammatory markers, and macrophage polarization-related factors after treatment with different hydrogel formulations. (**C**) Immunohistochemical staining of wound tissues showing the expression of MMP-9, TNF-α, IL-6, and IL-10 after treatment. EGCG, epigallocatechin gallate; GH, hydrogel; SGH, SBMA-containing hydrogel; Ca@Q-E, EGCG-loaded Ca-based oxygen-releasing nanoparticles. * *p* < 0.05 and *** *p* < 0.001 using one-way ANOVA followed by Tukey’s test. All values are mean ± SD. ns indicates no significant difference. All panels reproduced from [93] under the terms of the Creative Commons Attribution License. Copyright © 2025 The Author(s), published by Oxford University Press.

Wound-microenvironment-responsive polymers have been studied, including those responsive to pH, glucose, ROS, and hypoxia. For example, Liang et al. used immunometabolism-controlling small-molecule Metformin-loaded PBA/benzaldehyde bifunctional PEG-*co*-poly(glycerol sebacic acid) grafted chitosan hydrogel dressings [92]. The Schiff base bond in the benzaldehyde group with the amine group in chitosan, and the phenylboronate ester bond in the phenylboronic acid group with the catechol group, are cleaved by low pH and high glucose, respectively. Metformin released from chitosan hydrogel significantly reduced inflammatory cell number in wound sites. Similarly, Li et al. developed epigallocatechin gallate (EGCG) linked PBA-modified quaternary ammonium chitosan, which is responsive to ROS and glucose in diabetic wounds (Figure 5A) [93]. EGCG released from the hydrogel by ROS and glucose reprogrammed M1 macrophages into M2 macrophages. EGCG promoted the ERK signaling pathway (Figure 5B), which showed a 2.5-fold increase in CD206^+^ M2 macrophages and the decrease in inflammatory cytokine expression in wound sites (Figure 5C).

### 4.6. ROS-Scavenging Polymer for Macrophage Modulation

ROS scavenging characteristics are often observed in metal oxide nanoparticles and nanozymes [94,95,96,97,98]. In these approaches, hydrogels have generally been used as a local reservoir for ROS-scavenging nanomaterials within the wound bed, thereby improving their retention and reducing rapid loss from the wound site. Metal oxide nanoparticles or nanozymes can be incorporated by direct embedding within hydrogel networks, physical entrapment within porous hydrogel structures, or encapsulation in secondary nanoparticle carriers before hydrogel loading. Many studies have been focused on the integration of these nanomaterials with a hydrogel. He et al. fabricated a GelMA-cationic quaternary ammonium salt hydrogel containing ROS-scavenging CeO_2_ nanoparticles that carry protein kinase inhibitor Y-27632 [99]. The hydrogel exhibited antimicrobial properties, resulting in 99.99% antimicrobial activity against *S. aureus* and *E. coli*. ROS scavenging reduced the cGAS-STING pathway, triggering M2 polarization of macrophages as well. Together, hydrogel implantation into diabetic wound sites increased CD206^+^ M2 macrophages 3-fold compared to the control group.

Pu et al. used methacrylic anhydride-modified hyaluronic acid (AHAMA) hydrogel with chitosan nanoparticles [100]. Chitosan nanoparticles encapsulated polymetallic oxonate nanozyme and glucose oxidase. AHAMA hydrogel facilitated a sustained release of both nanozymes, which leads to glucose consumption, ROS scavenging, angiogenesis, and collagen regeneration. ROS scavenging upregulated the MAPK and Jun signaling pathway, expressing high levels of VEGF and IL4, resulting in M2 polarization in vitro. Fourteen days after implantation into diabetic wounds, 80% residual wound area was decreased compared to the untreated group.

### 4.7. Polymer-Based Gene Delivery Strategies for Macrophage Reprogramming

Macrophage reprogramming has been widely explored as an immunomodulatory strategy to promote wound healing. Gene delivery provides a direct strategy to reprogram macrophage phenotype by modulating intracellular transcriptional and inflammatory pathways, including IRF5, TLR4/NF-κB, and JAK/STAT activation [101,102,103,104,105]. In polymer-based systems, nucleic acid cargos, including siRNA, miRNA, mRNA, and plasmid DNA can be delivered to macrophages within the wound microenvironment. For example, Sharifiaghdam et al. developed chitosan-PEI nanocomplex for IRF5-siRNA delivery [101]. Layer-by-layer formulated chitosan-PEI-siRNA nanocomplexes were internalized into macrophages and exhibited endosomal escape, reducing M1 macrophage marker iNOS and increasing M2 macrophage marker Arg1. They then confirmed that nanocomplex-induced M2 macrophages promoted the migration of fibroblasts in vitro.

Saleh et al. utilized miRNA to drive the polarization of macrophages [103]. They incorporated HA-PEI-PEG-miR-223 nanoparticles into adhesive GelMA hydrogel. Nanoparticle-loaded hydrogel exhibited sustained release of miR-223 up to 48 h and upregulated Arg1 and downregulated iNOS and TNFα in macrophages in vitro. They demonstrated that Arg1/iNOS-positive cell ratio was significantly decreased in the wound tissue area 2 and 5 days after injury, resulting in wound closure acceleration.

### 4.8. Extracellular Vesicle-Loaded Polymeric Hydrogels for Macrophage-Mediated Repair

EVs, particularly mesenchymal or adipose stem cell-derived EVs, have been explored as immunomodulators and tissue repair mediators. Despite their advantages, naked EVs are rapidly cleared from wound sites and exhibit low local retention [106,107,108]. Polymer-based biomaterials could overcome these limitations by serving as depots that protect EVs. Li et al. developed mesenchymal stem cell (MSC)-derived EVs-loaded GelMA microspheres as a wound dressing [109]. GelMA microspheres facilitated stable lyophilization of MSC-EVs and sustained release of MSC-EVs from GelMA after rehydration. As a result, IL6 and IL10 expression levels in wound sites decreased and increased, respectively, 7 days after implantation of the GelMA microsphere in a diabetic foot injury. Interestingly, Peng et al. covalently bound adipose-derived mesenchymal stem cell (ADSC)-derived EVs with chitosan-grafted-dihydrocaffeic acid-based hydrogel [110]. In addition, they incorporated PF127 and tannic acid to form reversible interaction between the phosphate group of ADSC-EVs and the polyphenol group in the hydrogel. Sustained release of ADSC-EVs from the hydrogel over 15 days facilitated M2 macrophage polarization in vitro and a higher wound closure rate 14 days after implantation into the skin defect.

### 4.9. Macrophage Polarization Using Physical Cues

In addition to biological pathways that can modulate macrophage phenotypes, several studies have attempted to modulate macrophages using physical/mechanical cues. Many studies have shown that cytoskeletal changes in macrophages affect their phenotypes. M1 macrophages show a rounded shape, whereas M2 macrophages show an elongated shape [111,112,113]. Leveraging mechanotransduction of macrophage phenotypes, researchers have utilized micro- and nano-patterned polymers to change inflammatory activity in wound healing. For example, Xie et al. confirmed that aligned electrospun poly(L-lactide) nanofiber could control macrophage polarization [114]. Aligned fiber with a 758 ± 102 nm diameter and 5 MPa modulus significantly increased the expression level of Arg1 and deactivated the STAT1 pathway. They fabricated aligned fiber as a membrane platform and implanted it into skin defects in a mouse model. The number of CD206^+^ M2 macrophages was increased 2-fold compared to the unaligned fiber membrane. As a result, 90% of the residual wound area was reduced by the aligned fiber membrane at day 7.

Zhang et al. demonstrated that a conductive nanofibrous scaffold with electrostimulation could polarize macrophages for wound healing [115]. They fabricated an electrospun PU scaffold with a nanofibrous architecture and mixed it with carbon nanotubes (CNTs). The PU/CNT scaffold with 200 nm diameter showed high conductivity. Electrical stimulation (100 mV/cm, 10 min per day) after implantation into wound sites significantly increased wound closure rate, which is attributed to cytokine changes, including reduced TNFα and increased IL10, released from macrophages.

**Figure 6:**
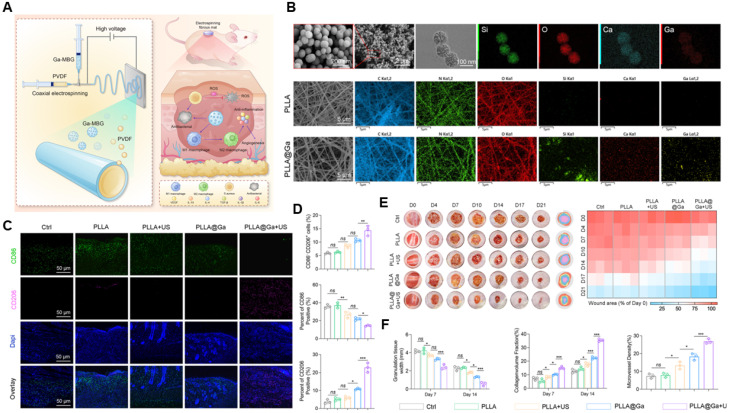
**Gallium-containing coaxial electrospun fibrous scaffold for infected wound repair.** (**A**) Schematic illustration of the Ga-MBG/PVDF coaxial electrospinning strategy and the proposed therapeutic effects of the resulting scaffold, including antibacterial activity, ROS regulation, macrophage polarization, and angiogenesis promotion. (**B**) Morphological and elemental characterization of Ga-MBG particles and PLLA@Ga fibrous scaffolds. (**C**) Immunofluorescence staining of wound tissues showing macrophage phenotype-related markers after different scaffold treatments. (**D**) Quantitative analysis of macrophage polarization markers, including CD68^+^CD206^+^ cells and CD86/CD206 expression patterns. (**E**) Representative wound images and heatmap-based wound area analysis showing accelerated wound closure after treatment with Ga-containing and ultrasound-assisted scaffold groups. (**F**) Quantitative histological analysis of granulation tissue formation, collagen deposition, and microvessel density during wound repair. * *p* < 0.05, ** *p* < 0.01, and *** *p* < 0.001 using one-way ANOVA followed by Tukey’s test. All values are mean ± SD. ns indicates no significant difference. All panels reproduced from [116] under the Creative Commons Attribution-NonCommercial-NoDerivatives 4.0 International License. Copyright © 2025 The Author(s), published by Springer Nature.

Interestingly, Liu et al. synthesized a multifunctional PLA electrospun dressing with gallium-doped mesoporous bioactive glass that could provide piezoelectric/electroactive cues to macrophages (Figure 6A,B) [116]. Electrical signals transformed from ultrasound significantly inhibited MAPK, NF-κB, and Toll-like receptor signaling pathways. Ultrasound stimulation after the implantation of PLA dressing reduced over 50% of CD86^+^ M1 macrophages, whereas CD206^+^ M2 macrophages were increased 5-fold compared to the untreated group (Figure 6C,D), resulting in near-complete wound closure, and increased granulation, collagen deposition, and blood vessel formation (Figure 6E,F).

Collectively, these studies indicate that monocyte- and macrophage-modulating polymeric materials should aim to restore macrophage plasticity rather than merely increase M2 marker expression. In chronic wounds, excessive monocyte recruitment and persistent inflammatory macrophage activation sustain cytokine production, oxidative stress, and matrix degradation, whereas timely reparative macrophage functions support efferocytosis, angiogenesis, fibroblast activation, collagen remodeling, and re-epithelialization. Polymer-based systems address these barriers by regulating monocyte trafficking, capturing inflammatory chemokines, delivering phenotype-modulating cargos, buffering ROS, incorporating EV- or gene-based signals, and presenting physical cues to guide macrophage behavior. Thus, the polymer matrix functions not only as a delivery vehicle but also as an immune-instructive microenvironment that controls local retention, release kinetics, cell–material interactions, and stage-specific repair signaling.

## 5. Conclusions and Future Perspectives

Polymer-based immunomodulatory materials have expanded the role of wound dressings from passive protection to active regulation of the immune microenvironment. Chronic and diabetic wounds are not simply delayed versions of acute wounds, but pathological inflammatory niches characterized by persistent neutrophil activation, excessive NET formation, sustained monocyte recruitment, macrophage polarization imbalance, oxidative stress, infection-driven stimulation, and impaired transition to tissue repair. The studies reviewed here demonstrate that polymeric materials can intervene in these processes through multiple mechanisms, including sustained release of immunomodulatory cargos, scavenging of inflammatory mediators, regulation of cell recruitment, delivery of nucleic acids or extracellular vesicles, and presentation of physical or electroactive cues.

A key conclusion is that neutrophils and macrophages should not be viewed only as inflammatory cells to be suppressed. Neutrophils are required for early host defense and wound debridement, but their persistent infiltration, oxidative burst, protease release, and NET accumulation can amplify tissue damage in chronic wounds. Accordingly, polymeric systems have been designed to reduce excessive neutrophil infiltration, redirect neutrophils toward reparative phenotypes, inhibit NETosis, degrade or scavenge preformed NETs, or, in infected wounds, enhance NET-mediated antibacterial activity in a controlled manner. Similarly, monocyte/macrophage-targeted materials should not simply eliminate inflammation. Their therapeutic value depends on restoring the proper sequence of monocyte recruitment, macrophage efferocytosis, inflammatory resolution, angiogenic support, collagen remodeling, and re-epithelialization. The polymer component is central to these strategies. Hydrogels, nanofibers, microneedles, microspheres, films, and hybrid nanocomposites determine whether immunomodulatory signals are retained locally, released at appropriate rates, protected from degradation, and spatially presented within the wound bed. Natural polymers such as chitosan, alginate, gelatin, hyaluronic acid, silk fibroin, and collagen provide biocompatibility and matrix-like interactions, whereas synthetic polymers such as PEG, PLGA, PLA, PCL, polyurethane, and functional polymer networks provide tunable degradation, mechanics, reproducibility, and scalable processing. In addition, polymer–nanozyme, polymer–EV, polymer–lipid, polymer–ion, and polymer–conductive material hybrids broaden the therapeutic space by integrating antioxidant, antibacterial, angiogenic, and immunoregulatory functions into a single dressing platform.

Future studies should move beyond reporting accelerated wound closure as the primary endpoint. Many polymeric wound materials show improved wound area reduction, collagen deposition, or histological regeneration, but the causal relationship between material properties and immune remodeling is often insufficiently defined. More rigorous temporal immune profiling is needed to determine how a material changes neutrophil activation, NET burden, monocyte recruitment, macrophage phenotype, efferocytosis, inflammatory cytokine networks, bacterial burden, ROS levels, angiogenesis, and matrix remodeling over time. In particular, macrophage modulation should be evaluated beyond simple M1/M2 marker expression, and neutrophil modulation should distinguish between beneficial early defense, pathological persistence, NET-associated damage, and reparative neutrophil phenotypes.

Another important direction is stage-adaptive material design. Chronic wounds are dynamic environments, and the same therapeutic cue is unlikely to be beneficial throughout all stages of repair. Early intervention may require antibacterial activity, biofilm control, ROS buffering, and limitation of excessive neutrophil-mediated damage, whereas later phases may require macrophage resolution, angiogenic support, fibroblast activation, epithelial migration, and matrix maturation. Therefore, future polymer systems should be designed to provide sequential, feedback-responsive, or microenvironment-triggered functions rather than indiscriminate multifunctionality. Stimuli-responsive hydrogels, multilayer dressings, core–shell fibers, microneedle depots, and hydrogel–nanoparticle composites are particularly suitable for this purpose.

Translation will also require a more balanced approach to material complexity. Highly engineered systems that combine multiple cargos, nanoparticles, responsive chemistries, and biological components may provide strong preclinical efficacy, but they also create challenges in sterilization, storage, batch reproducibility, degradation-product safety, regulatory classification, and cost. For clinical wound care, the most promising systems may not be the most complex ones, but those that combine a limited number of mechanistically justified functions with robust manufacturability and practical handling. Future research should therefore evaluate not only immune and regenerative outcomes, but also wet adhesion, dressing removal, repeated application, long-term biocompatibility, sterilization compatibility, and compatibility with existing wound-care workflows.

For clinical translation, however, polymer-based immunomodulatory wound materials must be evaluated not only for biological efficacy but also for manufacturability, sterilization compatibility, shelf-life, batch reproducibility, cost, and regulatory feasibility. These challenges are platform-dependent: nanoparticles require control of physicochemical properties and drug loading, hydrogels require stable and reproducible network formation, nanofibers require scalable fabrication and uniform drug distribution, and hybrid systems require additional safety assessment for multi-component interactions and long-term material retention. Translational considerations are summarized in Table 4.

In conclusion, polymer-based immunomodulatory wound materials should be designed as immune-instructive interfaces that coordinate inflammatory resolution with tissue reconstruction. Their success will depend on matching material function to wound type, immune stage, and pathological microenvironment. By integrating mechanism-guided immune profiling with clinically realistic polymer design, these systems have the potential to shift chronic wound therapy from passive coverage and nonspecific anti-inflammation toward active, stage-specific, and regenerative immune modulation.

## Figures and Tables

**Table 4:** Translational considerations for major classes of polymer-based immunomodulatory wound materials.

Material Platform	Major Translational Hurdles	Translational/Design Considerations	References
Polymeric nanoparticles	Batch reproducibility, manufacturing scale-up, retention, regulatory complexity	Standardized in PDI, surface charge, encapsulation efficiency, release profile, storage stability	[117,118,119]
Hydrogels	Sterilization compatibility, shelf-life, network reproducibility, cargo stability	Sterilization-compatible chemistries, reproducible crosslinking, lyophilized or precursor-based storage formats, validated release stability	[32,34]
Electrospun nanofibers	Scalable fabrication, uniform morphology, solvent residue control, reproducible drug loading	Process-controlled electrospinning, solvent removal validation, standardized fiber morphology, and release-profile QC	[37,120,121]
Hybrid systems	Multi-component reproducibility, safety, regulatory classification, long-term stability	Heterogeneity, complex degradation behavior, difficult large-scale production	[122,123,124]

## Data Availability

No new data were created in this study.

## Abbreviations

The following abbreviations are used in this manuscript:

ADSC	Adipose-derived mesenchymal stem cell
AHAMA	Methacrylic anhydride-modified hyaluronic acid
Arg1	Arginase 1
CNT	Carbon nanotube
ECM	Extracellular matrix
EGCG	Epigallocatechin gallate
EVs	Extracellular vesicles
GAG	Glycosaminoglycan
GelMA	Gelatin methacryloyl
HA	Hyaluronic acid
IL	Interleukin
iNOS	Inducible nitric oxide synthase
MPO	Myeloperoxidase
MSC	Mesenchymal stem cell
NETs	Neutrophil extracellular traps
NF-κB	Nuclear factor kappa B
PBA	Phenylboronic acid
PCL	Polycaprolactone
PCNs	Polyelectrolyte complex nanoparticles
PDGF	Platelet-derived growth factor
PEG	Polyethylene glycol
PEI	Polyethyleneimine
PGE2	Prostaglandin E2
PLA	Polylactic acid
PLGA	Poly(lactic-co-glycolic acid)
PPGA	Poly(PEGMA-co-GMA-co-AAM)
PU	Polyurethane
ROS	Reactive oxygen species
TNFα	Tumor necrosis factor alpha
VEGF	Vascular endothelial growth factor
